# HOXA5 Inhibits Metastasis via Regulating Cytoskeletal Remodelling and Associates with Prolonged Survival in Non-Small-Cell Lung Carcinoma

**DOI:** 10.1371/journal.pone.0124191

**Published:** 2015-04-14

**Authors:** Chi-Chung Wang, Kang-Yi Su, Hsuan-Yu Chen, So-Yi Chang, Chi-Fan Shen, Chia-Hung Hsieh, Qi-Sheng Hong, Ching-Cheng Chiang, Gee-Chen Chang, Sung-Liang Yu, Jeremy J. W. Chen

**Affiliations:** 1 Graduate Institute of Basic Medicine, Fu Jen Catholic University, New Taipei, Taiwan; 2 Department of Clinical and Laboratory Sciences and Medical Biotechnology, National Taiwan University College of Medicine, Taipei, Taiwan; 3 NTU Center of Genomic Medicine, National Taiwan University College of Medicine, Taipei, Taiwan; 4 Institute of Statistical Science, Academia Sinica, Taipei, Taiwan; 5 Institute of Biochemistry and Molecular Biology, National Taiwan University College of Medicine, Taipei, Taiwan; 6 Graduate Institute of Basic Medical Science, China Medical University, Taichung, Taiwan; 7 Division of Chest Medicine, Department of Internal Medicine, Taichung Veterans General Hospital, Taichung, Taiwan; 8 Department of Laboratory Medicine, National Taiwan University Hospital, Taipei, Taiwan; 9 Department of Pathology and Graduate Institute of Pathology, National Taiwan University College of Medicine, Taipei, Taiwan; 10 Center for Optoelectronic Biomedicine, College of Medicine, National Taiwan University College of Medicine, Taipei, Taiwan; 11 Institute of Biomedical Sciences, National Chung Hsing University, Taichung, Taiwan; 12 Agricultural Biotechnology Center, National Chung Hsing University, Taichung, Taiwan; University of South Alabama Mitchell Cancer Institute, UNITED STATES

## Abstract

Homeobox genes comprise a family of regulatory genes that contain a common homeobox domain and act as transcription factors. Recent studies indicate that homeobox A5 (HOXA5) may serve as a tumour suppressor gene in breast cancers. However, the precise role and the underlying mechanism of HOXA5 in lung cancer remain unclear. Oligonucleotide microarrays and an invasion/metastasis lung adenocarcinoma cell line model were used to determine the correlation between HOXA5 expression and cancer cell invasion ability. We found that ectopic expression of HOXA5 in highly invasive cancer cells suppressed cell migration, invasion, and filopodia formation *in vitro* and inhibited metastatic potential *in vivo*. Knockdown of HOXA5 promoted the invasiveness of lung cancer cells. In addition, HOXA5 expression was associated with better clinical outcome in non-small cell lung cancer patients with wild-type EGFR. Furthermore, genome-wide transcriptomic and pathway analyses were performed to identify the potential molecular mechanisms. Our data showed that HOXA5 may bind to the promoters of the cytoskeleton-related genes and downregulate their mRNA and protein expression levels. Our studies provide new insights into how HOXA5 may contribute to the suppression of metastasis in lung cancer via cytoskeleton remodelling regulation. Therefore, targeted induction of HOXA5 may represent a promising approach for non-small-cell lung cancer therapy.

## Introduction

Homeobox genes comprise a family of regulatory genes that contain a common 183-nucleotide sequence (homeobox) and encode specific nuclear proteins (homeoproteins) that act as transcription factors [1]. The homeobox sequence itself encodes a 61-amino acid domain, the homeodomain (HD), which is responsible for recognizing and binding to sequence-specific DNA motifs [2]. The specificity of this binding allows homeoproteins to activate or repress the expression of downstream effector target genes [3]. Class I homeobox genes, also termed *Hox* genes, are structurally and functionally homologous to the homeotic complex (HOM-C) genes of *Drosophila* [4]. The human genome encodes at least 39 homeobox genes organised in four clusters (A, B, C, and D), which are located on chromosomes 7, 17, 2, and 12, respectively [5].

During the last several decades, homeobox gene expression has been characterised in normal tissues and malignant cells and in the context of different diseases and metabolic abnormalities [6]. The HOX family genes play fundamental roles in the morphogenesis of vertebrate embryonic cells, providing regional information along the main body axis [7,8]. In addition, HOX genes have been implicated in angiogenesis and wound repair [9], in the function of the female reproductive tract [10], and in pulmonary hypertension and emphysema [11]. Because cancer and normal development have a great deal in common, as both processes involve shifts between cell proliferation and differentiation, mutations in or changes in the expression of homeobox genes are observed in many cancers, including leukaemia, colon, skin, prostate, breast, and ovarian cancers [12]. However, the functional relationships between the differential expression of homeobox genes and neoplastic phenotypes remain unclear.

One recent study showed that the expression of HOXA5 is lost in more than 60% of breast cancer cell lines and primary carcinomas due to promoter hypermethylation [13]. In addition, HOXA5 promoted breast cancer cell death through p53-dependent or caspase 2- and 8-activated apoptosis [13,14]. Furthermore, the loss of HOXA5 expression could lead to the functional activation of Twist, resulting in aberrant cell cycle regulation and the promotion of breast tumorigenesis [15]. Taken together, the data from these studies indicated that HOXA5 may serve as a tumour suppressor gene in breast cells. Several studies have investigated HOXA5 gene expression in human lung cancers [16–19]; however, the results of these studies are contradictory. Two reports showed that HOXA5 gene expression is downregulated by aberrant promoter methylation in the vast majority of non-small-cell lung cancers (NSCLCs) and that it may play an important role in the carcinogenesis of NSCLCs [16,17]. Nevertheless, the specific role and the underlying mechanisms of HOXA5 in lung cancer remain unknown. The objective of this study was to investigate the biological functions of HOXA5 in human lung adenocarcinoma cells and its association with survival in NSCLC patients.

## Materials and Methods

### Cell culture and patient specimens

Human lung adenocarcinoma cell lines, CL1-0, CL1-1, and CL1-5 (in ascending order of invasive competence) were established in our previous study [20]. All cell lines, including A549 (ATCC CCL-185), NCI-H322M obtained from National Cancer Institute, and PE089 [21], were maintained at 37°C in a humidified atmosphere containing 5% CO_2_. Cells were cultured in DMEM or RPMI 1640 medium (Life Technologies, Rockville, MD, USA) supplemented with 10% heat-inactivated fetal bovine serum (FBS; Life Technologies) and 1% penicillin-streptomycin (Life Technologies). Lung tumour tissue specimens were obtained from 68 patients with histologically confirmed NSCLC who underwent surgical resection at the Taichung Veterans General Hospital between September 2001 and May 2009. None of the patients had received pre-operative adjuvant chemotherapy or radiation therapy. This investigation was approved by the Institutional Review Board of the Taichung Veterans General Hospital (IRB No: CF13083). Written informed consent was obtained from all patients. The post-surgical pathologic stage of each tumour was determined according to the international TNM classification [22].

### 5-aza-2’-deoxycytidine treatment

Cells (5 × 10^5^) were seeded onto 15 cm dishes. After 24 hr, the cultured cells were washed with PBS and incubated in new medium containing 1 μM 5-aza-2’-deoxycytidine (5-aza-dC) (Sigma-Aldrich, St Louis, MO, USA). Every 24 hr, the incubated medium was refreshed with new medium containing the same concentration of 5-aza-dC. Total RNA was extracted from the treated cells using the Trizol reagent (Life Technologies) at day 5.

### Cell proliferation assay

Cells from each clonal line (CL1-5, Mock mix, HOXA5 mix, HOXA5 H8, H9, and H14) were seeded onto 96-well plates (3 × 10^3^ cells/well, 1 plate per cell line). After culturing for various durations, cell proliferation activity was evaluated by thiazolyl blue tetrazolium bromide (MTT) assay according to the manufacture’s protocol (Sigma-Aldrich Corp., St. Louis, MO, USA). Experiments were performed three times in triplicate.

### Oligonucleotide microarray analysis

The cRNA preparation and array hybridisation were performed as recommended in the Affymetrix GeneChip Expression Analysis Technical Manual. Briefly, 8 μg of total RNA was reverse-transcribed by a T7-(dT)24 primer (One-cycle cDNA Synthesis kit; Affymetrix, Santa Clara, CA). The cDNA product was then purified and transcribed in vitro with biotin-labelled ribonucleotides (IVT Labeling Kit; Affymetrix, Santa Clara, CA). A part of the biotinylated RNA molecules was fragmented and hybridized overnight to the Human Genome U133 Plus 2.0 GeneChip (Affymetrix, Santa Clara, CA). Finally, the GeneChip was washed and developed by the amplification staining protocol provided by Affymetrix. The GeneChip was scanned using an Affymetrix GeneChip Scanner 3000 7G, and the images were extracted with Affymetrix GeneChip Operating Software (GCOS) version 1.4. All microarray experiments were performed in triplicate with cRNA probes prepared from HOXA5-transfected and mock-transfected CL1-5 cells. The microarray data were filtered by 2-fold change under FDR protection (P<0.05) using Genespring GX 11 (Silicon Genetics, Redwood, CA, USA). The details of these procedures and statistical analyses have been described previously [23]. The microarray data has been deposited to Gene Expression Omnibus (GEO) with deposit ID GSE50659. The results of the microarray analysis were confirmed with SYBR Green real-time RT-PCR.

### Real-time quantitative reverse transcription-PCR

The expression level of HOXA5 in clinical specimens was measured with quantitative Taqman real-time PCR in an ABI Prism 7900 sequence detection system (Applied Biosystems, Carlsbad, CA, USA). The primer set for HOXA5 (ID: Hs00430330_m1) was purchased from Applied Biosystems. To validate the levels of expression of genes found on microarray analysis, SYBR Green RT-PCR was performed. TATA-box binding protein (TBP) was used as an internal control (GenBank X54993). The relative expression level of HOXA5 compared with that of TBP was defined as -ΔCT = -[CT_HOXA5_-CT_TBP_]. The HOXA5/TBP mRNA ratio was calculated as 2^-ΔCT^ × K, in which K is a constant.

### Western blot analysis

Western blot analysis was used to examine the protein expression levels of HOXA5 in various cell lines. The details of these procedures were described previously [24]. The primary antibody against HOXA5 was purchased from Santa Cruz Biotechnology. α-tubulin was used as an internal control to ensure equivalent gel loading. After incubation with the primary antibodies, membranes were washed three times with TBST, followed by incubation with horseradish peroxidase (HRP)-conjugated secondary antibodies (Santa Cruz Biotechnology, Santa Cruz, CA, USA) and detection using an enhanced chemiluminescence detection system (ECL, GE Healthcare, Piscataway, NJ, USA).

### Invasion and migration assays

The invasiveness of CL1-5 cells transfected with HOXA5 or vector alone was examined in a transwell assay using chambers (8-μm pore size; Corning Costar, Cambridge, MA, USA) and transwell filters coated with Matrigel (BD Biosciences, Franklin Lakes, NJ, USA), as described previously [24]. 2× 10^4^ cells per well were seeded onto the upper wells of precoated transwells. After 18 hr of incubation, the number of cells attached to the lower surface of the polycarbonate filter was determined under a light microscope at 400× magnification. The migratory capability of the CL1-5 cells transfected with HOXA5 or vector alone was assessed using wound healing assays, as described previously [24]. The number of cells migrating into the cell-free zone was counted under a light microscope. All experiments were performed in triplicate.

### siRNA transient transfection

Desalted siRNA duplexes were synthesised by Qiagen (Qiagen, Crawley, UK) and were annealed according to a standard protocol. The HOXA5 and scrambled siRNAs were transfected using the RNAiFect Transfection Reagent (Qiagen) according to the manufacturer’s instructions. The efficacy of the siRNA was monitored by immunoblotting with a HOXA5 antibody. The invasiveness of CL1-0 cells transfected with HOXA5 siRNA or scramble siRNA was examined in a transwell assay as described previously [24].

### Single cell movement assay

Cells (5×10^3^) were seeded into six-well tissue culture dishes and cultured in medium containing 10% FBS and cellular debris were removed by washing with PBS. The cultures were incubated at 37°C and photographed immediately and at every 30 minutes for 24 hrs. The movement of each cell was analysed by measuring the distance travelled by the cell nucleus over 24 hr. To track the migration path of individual cells, cells were manually traced in consecutive frames, and the geographical centers were recorded using MetaMorph image analysis software (Molecular Devices, Sunnyvale, CA, USA). The migration rate of the cell was calculated as the ratio of the total length of the migration path to the duration of migration. The average migration speed in μm/hr was calculated by analysing at least 25 cells/group.

### Immunofluorescence and immunohistochemical staining

For immunofluorescence staining, cells were cultured on 12 mm glass coverslips, fixed for 15 min in 4% paraformaldehyde, permeabilised, and stained with primary antibodies (Fascin [ab49815]; Abcam, Cambridge, UK and Myosin-X [22430002]; Novus Biologicals, Littleton, CO, USA) followed by secondary Alexa 488-conjugated goat anti-mouse antibodies (Invitrogen, Carlsbad, CA, USA). TRITC-conjugated phalloidin was used to stain actin filaments. Images were acquired with a confocal microscope (Carl Zeiss, Oberkochen, Germany). Nuclei were counterstained with 4’, 6-diamino-2-phenylindole (DAPI). For immunohistochemical staining, the lungs of subcutaneously injected mice were removed, weighed, and fixed in 10% formalin. The embedded tissues were sliced into 4 μm sections and stained with hematoxylin-eosin or an anti-vimentin antibody for histological analysis. The brightness, contrast, colour balance, and final image size were adjusted using Adobe Photoshop CS2.

### *In vivo* experimental metastatic animal model

Six-week-old severe combined immunodeficiency (SCID) mice were purchased from the National Laboratory Animal Center of Taiwan. All animals were housed in an SPF environment, and all experiments were approved by the Board of Animal Welfare of the National Taiwan University College of Medicine. The intravenous injection of tumour cells was performed as described in a previous study [24]. Briefly, HOXA5-transfected or mock-transfected cells (1×10^6^) were resuspended in 0.1 ml PBS and injected into the tail vein of each mouse. At 28 days after the injection, the mice were sacrificed, and their lungs were harvested, fixed in Bouin’s solution and photographed. The numbers of metastatic tumour nodules in the lungs were counted under a dissecting microscope.

### Chromatin immunoprecipitation assays

A chromatin immunoprecipitation (ChIP) assay kit (Upstate Biotechnology, Lake Placid, NY, USA) was used according to the manufacturer’s instructions. Briefly, CL1-0 cells were cross-linked in a 1% formadehyde solution for 10 min. Cells were then lysed in SDS buffer and sonicated to generate 200–1,000 bp DNA fragments. After centrifugation, the supernatant was diluted with ChIP buffer and incubated with the indicated antibodies at 4°C overnight. Immune complexes were precipitated, washed, and eluted as recommended by the manufacturer. Samples were reverse cross-linked by heating at 65°C. DNA fragments were purified and an aliquot of each sample was used as the template for PCR analysis.

### Statistical analysis

All in vitro experiments were performed three times in triplicate, and significant differences were identified using ANOVA (Excel, Microsoft). Where appropriate, the results are presented as the means ± SD. For clinical investigation, we divided patients into high-expression and low-expression groups using the 70% percentile for the level of HOXA5 RNA as a cut-off point. The patient characteristics in the high-expression and the low-expression groups were compared using Student’s t-test for continuous variables or Fisher’s exact test for categorical variables. In the survival analysis, the overall survival curves were produced by the Kaplan-Meier method, and the log-rank test was used to test the differences between survival curves. All analyses were performed with SAS version 9.1 software (SAS Institute, Cary, NC, USA). All statistical tests were two-sided, and P values<0.05 were considered statistically significant.

## Results

### Correlation between the expression of HOXA5 and lung cancer cell invasion

**Fig 1:**
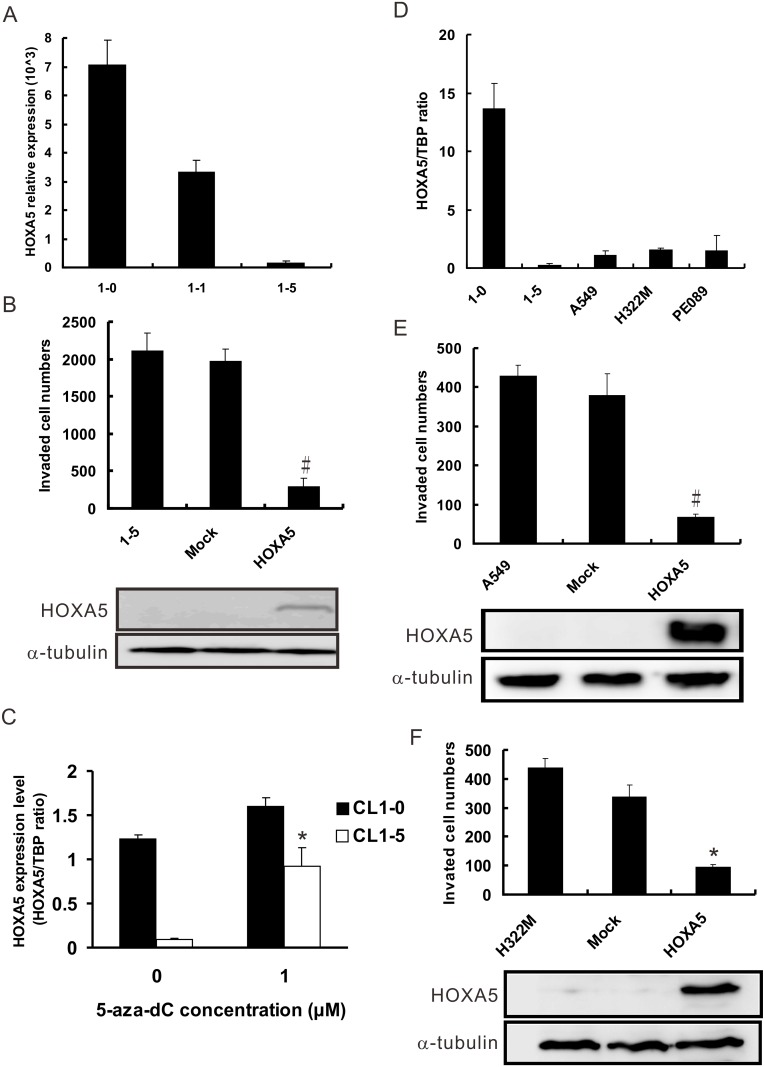
Negative correlation between NSCLC cell invasion and HOXA5 expression. (**A**) The mRNA expression level of HOXA5 in a lung adenocarcinoma cell line model, as assessed by microarray analysis. The invasive ability of the cell lines increased from CL1-0, CL1-1 to CL1-5. (**B**) The invasiveness of CL1-5 cells with transient HOXA5 expression and control cells (Mock) as evaluated by modified Boyden chamber assay. #, *P*<0.01 compared with control cells. (**C**) The effect of demethylation on HOXA5 expression. CL1-0 and CL1-5 cells were treated with 5-aza-2’-deoxycytidine for 5 days and subjected to QRT-PCR analysis. *, *P*<0.05 compared with untreated cells. TATA-binding protein (TBP) was used as an internal control. (**D**) The HOXA5 mRNA level in different NSCLC cells after 48 hr incubation was measured by QRT-PCR analysis. All experiments were performed three times in triplicate. *In vitro* invasion assays of low endogenous HOXA5-expressing A549 (**E**) and H322M (**F**) cells with transient HOXA5 overexpression and control cells (Mock). #, *P*<0.001; *, *P*<0.05 compared with the mock. TATA-binding protein (TBP) was used as an internal control in real-time RT-PCR assays. α-tubulin was used as a loading control in Western blot assays.

In our previous study, we screened a series of human lung adenocarcinoma cell lines with various invasive capabilities using microarray analysis and identified several hundred genes for which expression levels were associated with invasion [20]. Among these genes, we notice HOXA5 because of the dramatically differential expression between different cell lines. The expression of HOXA5 is inversely associated with cell invasive ability. The mRNA expression of HOXA5 was higher in the less invasive CL1-0 and CL1-1 cells than in the highly invasive CL1-5 cells, as indicated by the microarray analysis signal (Fig 1A). To further confirm that the expression of HOXA5 is correlated with the invasive capabilities of cells, the HOXA5-expressing vector was transiently transfected into a cell line with lower expression of endogenous HOXA5, CL1-5, and an *in vitro* invasion assay was performed to evaluate the impact of HOXA5 on invasion. The data showed that HOXA5 could suppress the invasive abilities of CL1-5 cells (Fig 1B). Because many previous reports have suggested that HOXA5 expression is affected by aberrant promoter methylation, we examined this possibility in our cell line model. We treated CL1-0 and CL1-5 cells with 1 μM of the demethylation agent 5-aza-dC and extracted the cellular mRNAs for Real-time quantitative reverse transcription-PCR (QRT-PCR) analysis. As shown in Fig 1C, the expression of HOXA5 in the high HOXA5-endogenous expressing and less invasive CL1-0 cells was not significantly affected by 5-aza-dC treatment. However, in the low endogenous expressing and highly invasive CL1-5 cells, treatment with 1 μM of 5-aza-dC caused an approximately nine-fold enhancement of HOXA5 expression (*P*<0.05 compared with the vehicle control). We then examined the expression of HOXA5 in other lung cancer cell lines, including A549, H322M, and PE089. The expression of HOXA5 was low in these tested lung cancer cell lines, except for CL1-0 cells (Fig 1D). To determine whether these phenomena are cell-specific, two additional cell lines, A549 and H322M, were transiently transfected with HOXA5-expressing or mock control plasmids and then tested by the invasion assays. The results showed that the transfectants with higher HOXA5 expression levels had significantly lower invasive capabilities than the mock transfectants in both A549 (Fig 1E) and H322M cells (Fig 1F).

### Overexpression of HOXA5 suppresses lung cancer cell migration and invasion *in vitro*

**Fig 2:**
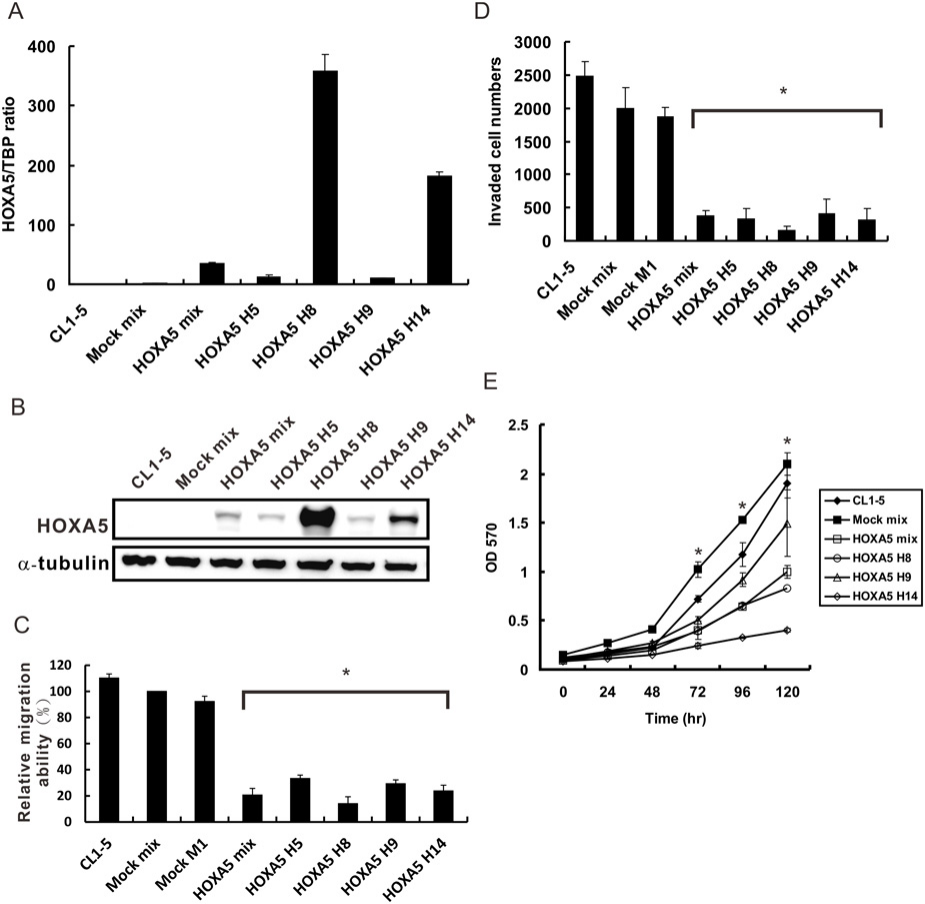
Suppression of NSCLC cell invasion and migration *in vitro* by enforced expression of HOXA5. (**A**) HOXA5 mRNA expression in transfected cells with constitutive HOXA5 expression, as measured by QRT-PCR analysis. The pcDNA3.1 vector transfectants were used as control cells (Mock mix). TBP was used as an internal control. (**B**) HOXA5 protein expression in the transfectants as determined by Western blot analysis with an anti-HOXA5 antibody; α-tubulin served as a loading control. (**C**) The cell migration ability of HOXA5 transfectants was assessed by scratch wound healing assays. The number of cells migrated into the cell-free zone was evaluated at 8 hr after wounding. The data are representative of three independent experiments and are shown as the mean ± SD. *, *P*<0.05, compared with the Mock mix. (**D**) The invasiveness of CL1-5, HOXA5 and mock transfectants was evaluated by transwell assays. Three independent experiments were performed. *, *P*<0.05 compared with the Mock mix. (**E**) The proliferation activity of CL1-5, HOXA5 and mock transfectants was examined by MTT assay. *, *P*<0.05 compared with the Mock mix.

To clarify the effects of HOXA5 expression on lung cancer progression, CL1-5 cell clones with stably constitutive HOXA5 expression were established. The mRNA and protein expressions of HOXA5 in these stable cell lines were measured using QRT-PCR and Western blot analyses, respectively (Fig 2A and 2B). The transfected cell clones expressed higher levels of HOXA5 mRNA and protein than did the mock control (Mock mix). Four single clones (HOXA5 H5, H8, H9, and H14) and one mixed clone (HOXA5 mix) that stably expressed HOXA5 were isolated for further studies. The migration ability of the HOXA5 transfectants in the wound healing assay was markedly reduced, to 14%-33%, compared with that of the mixed mock control (Fig 2C). To further confirm whether the invasive phenotype of the lung cancer cells was correlated with HOXA5 expression, as the microarray data suggested, the invasion assay was performed in these cell clones. The stably HOXA5-overexpressing cell lines showed significantly lower invasive capabilities than mock-transfected and CL1-5 cells (Fig 2D and S1 Fig). This result is consistent with that shown in Fig 1B. To rule out the possibility that the inhibitory effect of HOXA5 on lung adenocarcinoma cell migration and invasion capabilities is through proliferation blockage, MTT assay was performed. As shown in Fig 2E, highly expressed HOXA5 transfectants (HOXA5 H8 and H14) significantly reduced proliferation activity compared with mock-transfected and parental CL1-5 cells only after 48h.

### HOXA5 knockdown rescues the invasive capability of lung cancer cells

**Fig 3:**
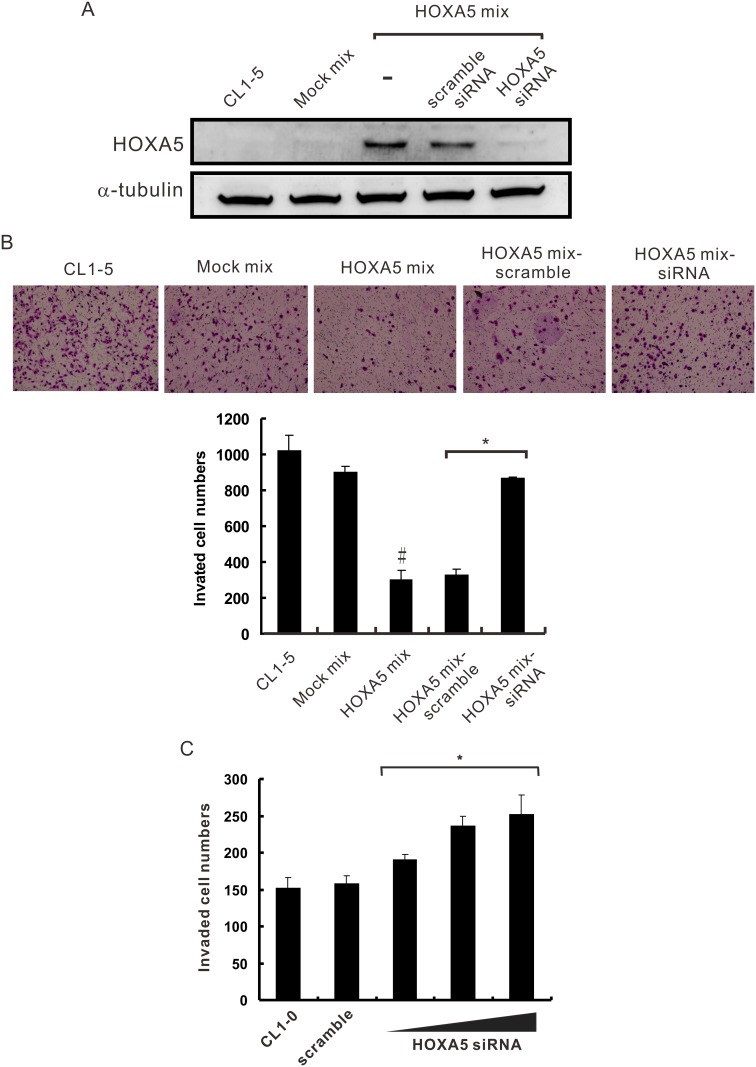
Enhancement of NSCLC cell invasiveness by silencing HOXA5 expression. (**A**) Silencing efficacy of the HOXA5-specific siRNA was determined by Western blot analysis using an anti-HOXA5 antibody; α-tubulin served as a loading control. (**B**) Constitutively HOXA5-overexpressing cells (HOXA5 mix; 1× 10^4^ cells/well) were treated with 20 nM of HOXA5-specific siRNA or scrambled siRNA and then subjected to invasion assays. Upper panel, the representative photographs of invasion assays. Lower panel, the invasive capability of HOXA5 transfectants was restored by HOXA5-specific siRNA treatment. #, *P*<0.01 compared with the Mock mix; *, *P*<0.05 compared with the scrambled siRNA control. (**C**) Silencing HOXA5 increased the invasiveness in a dose-dependent manner. CL1-0 cells were transfected with scramble siRNA (scramble) or various concentrations of HOXA5 siRNA and were assayed for invasiveness by transwell assays in triplicate. *, *P*<0.05 compared with the scramble siRNA control. The data are presented as the mean ± SD.

To confirm the functional consequence of reduced HOXA5 expression within lung cancer cells, we evaluated the ability of stable HOXA5-overexpressing mixed cell clone (HOXA5 mix) and CL1-0 cells with highly expressed HOXA5 to invade through Matrigel-coated transwell filter membranes following transfection with HOXA5 siRNAs. The HOXA5 protein expression in these HOXA5-silenced cells was markedly decreased compared with that in the transfection control and the scramble siRNA-transfected control and was almost equal to the HOXA5 levels of mock mixed clones and non-transfected CL1-5 cells (Fig 3A). Consistent with the results of the invasion assay described above, the transfection of scramble control siRNA did not influence the invasive capability of HOXA5-overexpressing cells (HOXA5 mix). However, the invasiveness of HOXA5 siRNA-transfected cells was nearly equivalent (96.2%) to that of the mock mixed clone (Fig 3B). Moreover, the expression levels of HOXA5 mRNA in HOXA5-silenced CL1-0 cells were dramatically reduced to 11% as compared to scramble control (S2 Fig). Endogenous HOXA5 knockdown increased CL1-0 cell invasion capabilities in a dose-dependent manner (Fig 3C). Taken together, these results indicate that the overexpression of HOXA5 could reduce lung cancer cell invasion and migration capabilities and re-silencing of HOXA5 is able to restore the invasive capability of these cells. These data imply that HOXA5 could act as a suppressor of invasion.

### HOXA5 suppresses migration rate and filopodia formation in lung cancer cells

**Fig 4:**
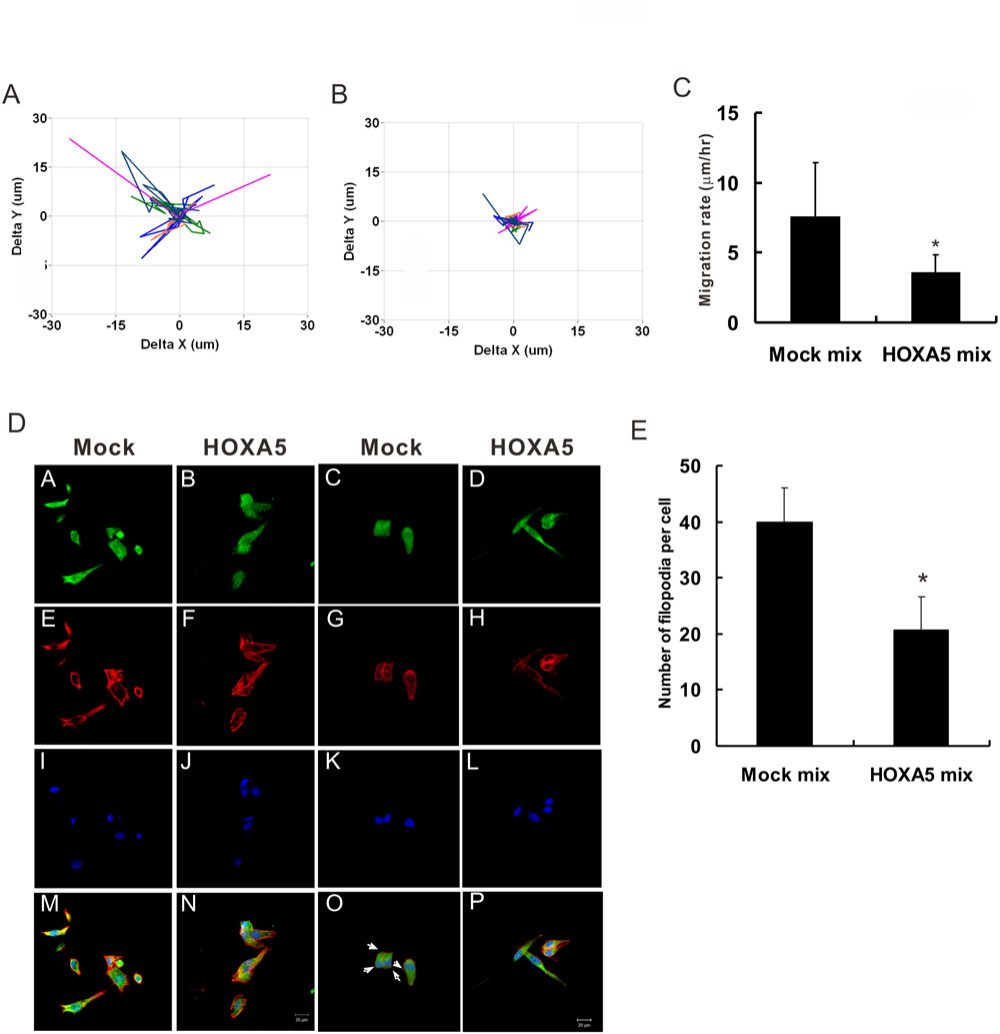
Suppression of NSCLC cell migration and filopodia formation by HOXA5. The migration of individual cells was monitored using time-lapse microscopy. The data are presented as overlays of representative trajectories of (**A**) mock control cells (Mock mix) and (**B**) HOXA5-overexpressing cells (HOXA5 mix). (**C**) The effect of HOXA5 on single cell migration. The median speed was calculated by tracking the movement of individual cells with around 25 cells per assay condition in three independent experiments. *, *P*<0.05 compared with the mock control. (**D**) Representative photographs of immunofluorescence staining of endogenous F-actin in stably expressing HOXA5 or control vector cells (Mock). Cells were fixed and stained for fascin (A, B), Myosin-X (C, D) and F-actin (E-H). Nuclei were counterstained with DAPI (I-L). Merge images were also shown (M-P). Scale bars, 20 μm. (**E**) The numbers of filopodia per cell were counted (n = 15 cells per group). Data are presented as the mean ± SD. *, *P*<0.05 compared with the mock control.

We next evaluated the function of HOXA5 in cell migration directly, which is the most critical step of tumour invasion and metastasis. The results of cell tracking assays showed that the overexpression of HOXA5 significantly decreased cell migration rates in highly migratory CL1-5 cells assayed in a single cell condition (Fig 4A–4C). F-actin reorganisation is known to play a critical role in filopodia formation and cell movement. To investigate whether the effect of HOXA5 on lung cancer invasion and migration is associated with actin filament dynamics, we examined the cellular location and distribution of actin filaments with confocal microscopy. Although the expression patterns of actin binding protein, fascin, showed no dramatic differences between HOXA5-overexpressing cells and mock control cells, the myosin-X proteins that expressed on filopodia tips were significantly decreased in HOXA5-overexpressing cells compared to mock control cells (Fig 4D). In addition, we found that HOXA5-overexpressing cells had fewer filopodia than did mock control cells (Fig 4E). Taken together, these results indicate that HOXA5 plays an important role in cell migration.

### HOXA5 suppresses metastasis *in vivo*

**Fig 5:**
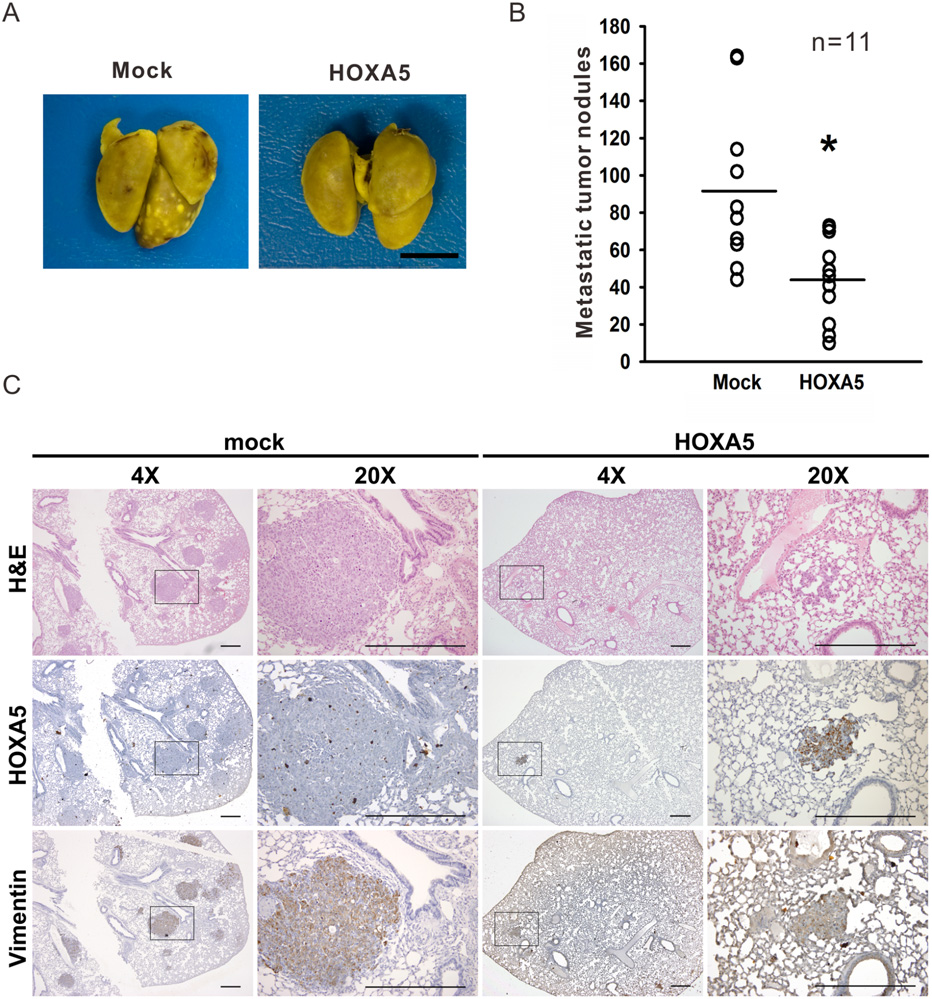
Inhibition of *in vivo* metastasis by HOXA5 overexpression. (**A**) A representative image of mouse lungs with tumour nodule formation. HOXA5- or mock-transfected CL1-5 cells (1×10^6^) were intravenously injected into the tail vein of the mouse. The lungs were harvested and fixed in Bouin’s solution 28 days after the injection. Scale bar, 5 mm. (**B**) HOXA5 overexpression reduced the number of lung metastatic tumour colonies. Mice injected with HOXA5-transfected tumour cells had fewer lung nodules (44.2 ± 6.9 nodules/mouse, n = 11) than mock-transfected mice (91.2 ± 12.4 nodules/mouse, n = 11). All data are shown as the mean ± SD. *, *P*<0.05 compared with the mock control. (**C**) Representative H&E and IHC staining of lung metastasis in each group. Human lung adenocarcinoma cells were stained with anti-human vimentin or anti-human HOXA5. Square frames in the low magnificent field were showed in high magnificent field. Scale bar, 200 μm.

To examine the role of HOXA5 in the antimetastatic process *in vivo*, an i.v. experimental lung metastasis model in SCID mice was used. HOXA5 mixed or mock mixed cell clones were injected into the tail veins of SCID mice. After twenty-eight days, the numbers of colonised pulmonary tumour nodules were counted. Most of the pulmonary tumour colonies in the mice were found near or on the surface of the lungs (Fig 5A). These data showed that the HOXA5-overexpressing cells produced fewer pulmonary tumour nodules than mock control cells (44.2 ± 6.9 vs. 91.2 ± 12.4 nodules per mice; *P*<0.05, n = 11 in both groups) (Fig 5B). In order to confirm the observed nodules were derived from our injected tumor cells, immunohistochemical staining was performed by utilizing anti-human vimentin as well as anti-HOXA5 antibodies (Fig 5C). These data indicate that HOXA5 may play a critical role in the antimetastatic process in lung cancer.

### HOXA5 expression and prognosis in non-small cell lung cancer patients

**Fig 6:**
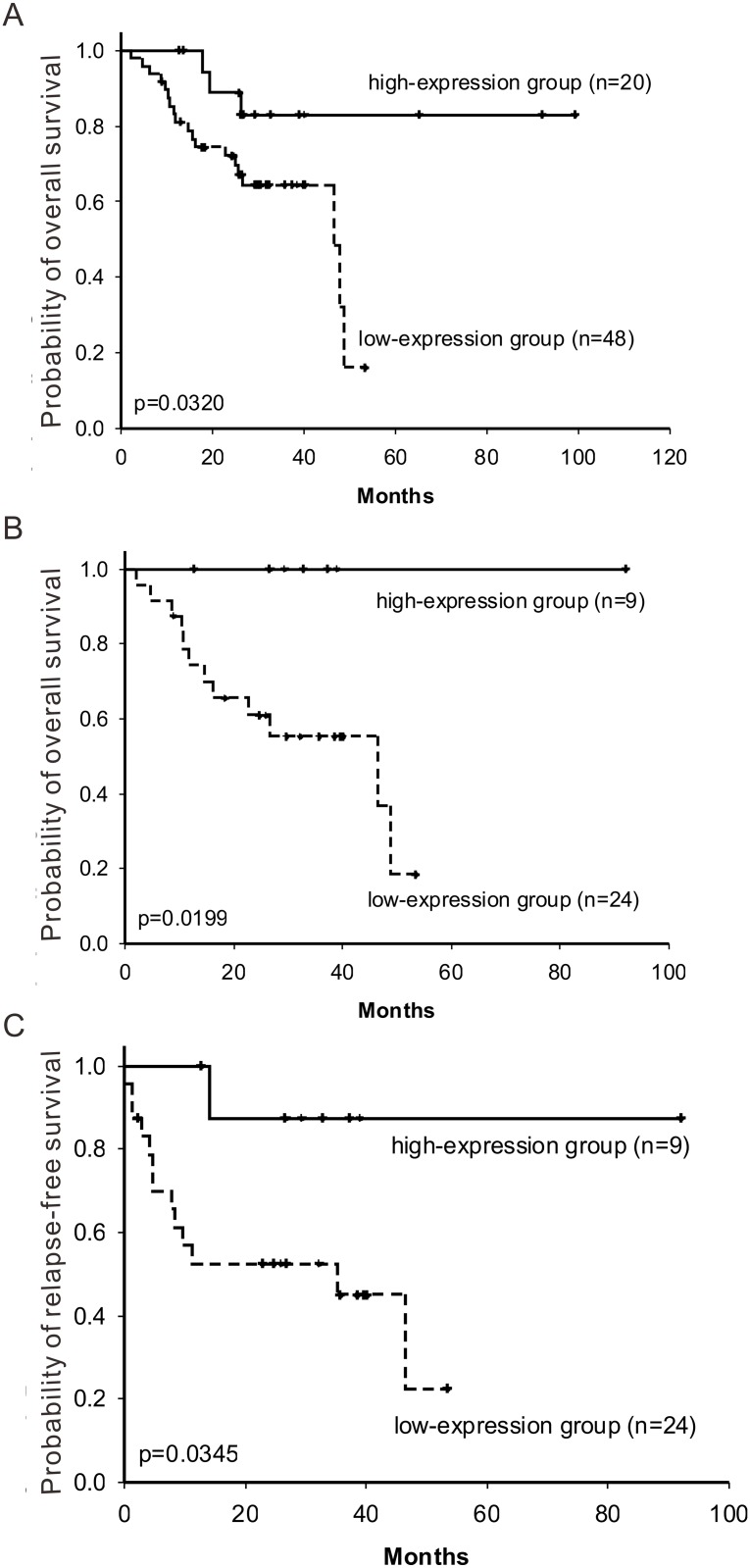
The effect of HOXA5 expression on the survival of NSCLC patients. (**A**) Overall survival of 68 NSCLC patients. (**B**) Overall survival and (**C**) relapse-free survival in the 33 NSCLC patients with the wild-type EGFR allele. The expression level of HOXA5 in this cohort was determined by real-time quantitative RT-PCR assays of patient tissue samples. The 70% percentile of the HOXA5 mRNA expression levels was used as the cut-off point between the high-expression and low-expression groups. A 2-sided log rank test was used to test for differences between groups.

The clinical characteristics of the included patients are shown in S1 Table. To elucidate the clinical relevance of HOXA5 in NSCLC patients, we analysed a cohort of 68 NSCLC specimens using QRT-PCR assay. Patients with high HOXA5 expression had longer overall survival than those with low expression (Fig 6A; *P* = 0.0320, log rank test), whereas the disease-free survival result was not significant. To rule out the effect of EGFR mutation on survival, a subset of NSCLC patients with wild-type EGFR was further selected and analysed for patient survival. The Kaplan-Meier survival analyses showed that HOXA5 expression is associated with the better overall survival and disease-free survival in NSCLC patients with the wild-type EGFR (Fig 6B and 6C; *P* = 0.0199 and 0.0345, respectively, log rank test).

### Identification of HOXA5 downstream target genes by microarray analysis

**Fig 7:**
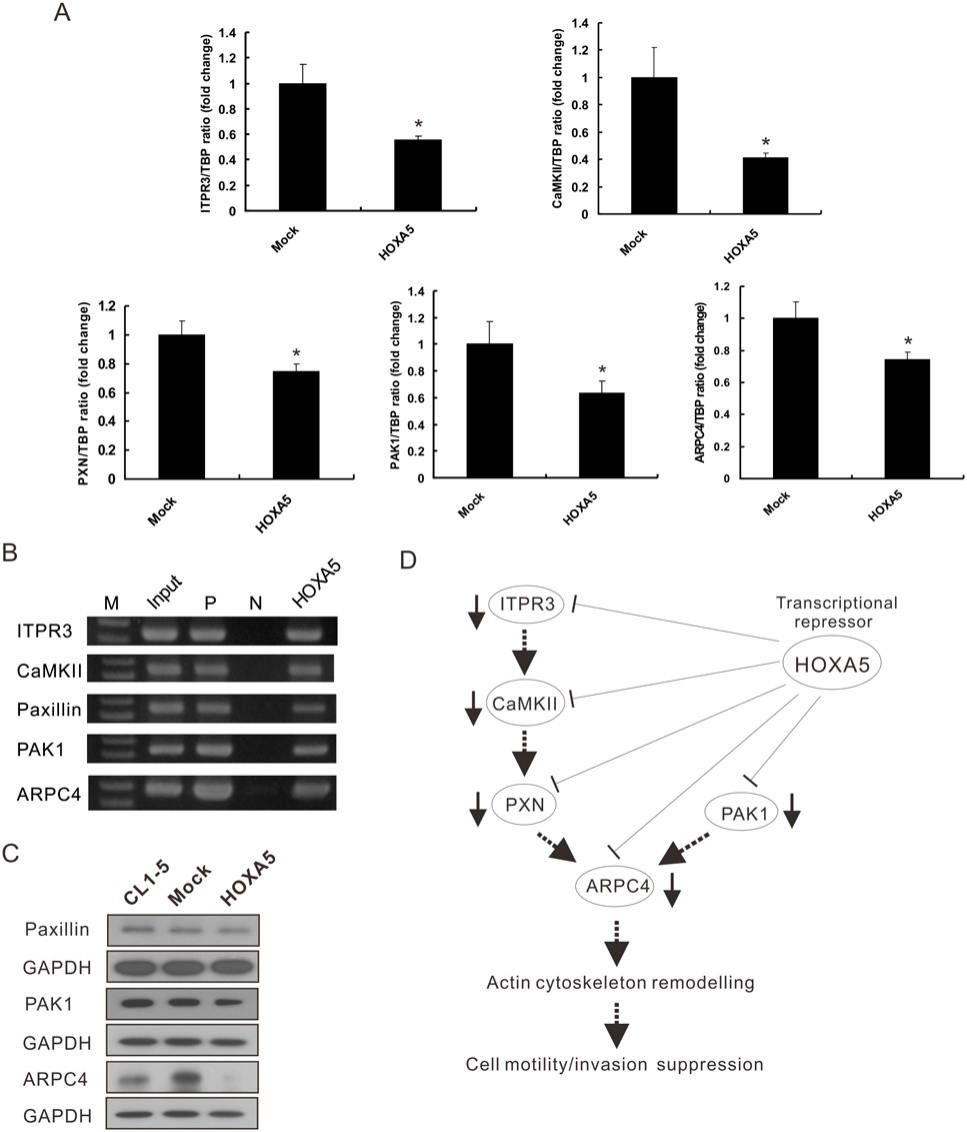
Regulation of actin cytoskeleton-related genes by HOXA5. (A) The mRNA expression levels of target genes in HOXA5 constitutively overexpressing cells (HOXA5) and pcDNA3.1 vector transfectants (Mock) were measured by quantitative RT-PCR. TBP was used as an internal control. The data are presented as the mean ± SD of the results from three independent experiments. *, *P*<0.05 compared with the mock control. (B) ChIP analysis was performed for *in vivo* binding of HOXA5 on the promoters of candidate genes. Sheared chromatin fragments were immunoprecipitated with the indicated antibodies, and the candidate genes promoter regions were amplified by PCR. M, 100-bp DNA marker; input, ChIP assay loading control; P, anti-acetyl Histone H3 Ab, positive control; N, rabbit normal IgG, negative control. (C) The expression levels of actin remodelling-related proteins in the CL1-5, Mock and HOXA5 transfectants were determined by Western blot analysis. GAPDH served as a loading control. (D) A proposed model of HOXA5-mediated cancer cell invasive suppression. In HOXA5-overexpressing cells, several actin remodelling-related genes were transcriptional downregulated by HOXA5 and resulted in the suppression of the migration and invasion capabilities.

To characterise the mechanisms through which HOXA5 inhibits cancer cell invasion and metastasis, Affymetrix oligonucleotide microarrays were employed to identify genes that were differentially expressed between HOXA5 transfectants and mock control cells. A total of 869 genes (t-test, FDR <0.05) with at least two-fold change in expression levels were identified and subjected to pathway analysis. The top 8 ranking pathways were listed in S2 Table, including cytoskeleton remodelling. A part of the differentially expressed genes related to cell invasion, movement, adhesion, and signal transduction were listed in S3 Table. Because the dynamic change of actin cytoskeleton is critical for invading and migrating cells [25] and microarray analysis also indicated that HOXA5 might alter the expression levels of genes associated to actin remodelling, we determined the expression levels of actin remodelling-related genes by SYBR-Green real-time RT-PCR (Fig 7A). These results showed that overexpression of transcription factor HOXA5 could significantly reduce the mRNA expression levels of paxillin (PXN), actin related protein 2/3 complex (ARPC4) and p21 protein-activated kinase 1 (PAK1). In addition, two genes which involved in the signaling transduction pathways, including calcium/calmodulin-dependent protein kinase II (CaMKII) and inositol 1,4,5-triphosphate receptor, type 3 (ITPR3), were also decreased under HOXA5 induction. Knockdown of HOXA5 expression by HOXA5-specific siRNA could reverse these effects (S3 Fig).

To further evaluate whether the transcription factor HOXA5 could directly interact with the promoters of these genes *in vivo*, the chromatin immunoprecipitation (ChIP) analysis with PCR primers that span the conserved HOXA5 binding sites within these promoters was performed. As shown in Fig 7B, the results indicated the binding of HOXA5 on the promoters of these five genes. Taken together, our results suggest that HOXA5 could directly regulate these genes in a transcriptional level and serves as a transcriptional repressor of these actin remodelling-related genes. Furthermore, the Western blot results also showed that overexpression of HOXA5 could decrease the protein expression levels of Paxillin, PAK1, and ARPC4 (Fig 7C). Based on these findings, we proposed a novel putative model of HOXA5-mediated suppression of NSCLC cell metastasis (Fig 7D).

## Discussion

HOXA5 has been known principally for its role in pattern formation during development [7,8]; however, several studies have suggested that it may also play a role in cancer cell migration, invasion and metastasis. The data presented here show that the overexpression of HOXA5 could suppress lung adenocarcinoma cell migration and invasion *in vitro* and *in vivo*. The knockdown of HOXA5 rescued the invasive capabilities of lung adenocarcinoma cells. In addition, increased levels of HOXA5 expression were associated with better overall and disease-free survivals in NSCLC patients with wild-type EGFR. ChIP and real-time RT-PCR results suggested that HOXA5 could serve as a transcriptional repressor of some actin remodelling-related genes. This is the first study to demonstrate that HOXA5 can inhibit NSCLC cell invasion and metastasis through the regulation of the actin cytoskeleton.

HOX genes are dysregulated in metastatic lesions relative to the primary tumour and the corresponding normal tissue, supporting the hypothesis that homeoproteins may be involved in cancer progression [26]. In some cases, HOX gene expression appears to be specifically linked to the stage of tumour progression and histological tumour types [27]. For instance, HOXB6, HOXB8 and HOXC9 are dysregulated at various stages of colon cancer development [28]. The loss of HOXA5 expression may result in aberrant homeostasis and tumorigenesis. A previous report indicated that the loss of p53 expression in human breast cancer may be primarily due to a lack of HOXA5 [13]. Our data shown in Fig 3A and 3B clearly demonstrated that HOXA5 siRNA can significantly knock down the overexpressed HOXA5 and relieve the suppression of invasion induced by overexpressed HOXA5 in CL1-5 cells. Although the invasion change is smaller in Fig 3C than in Fig 3B, it is important data to support knockdown of endogenous HOXA5 capable of promoting invasion in nature context. Moreover, invasion is a complicated cellular progress that many genes and mediators involved in. Hence, HOXA5 might be just one of genes that contribute to the lower invasiveness of CL1-0 compared with CL1-5. Silencing of HOXA5 might not overcome the invasion suppression controlled by other genes.

Two recent studies also showed that microRNA-196a and microRNA-130a could downregulate HOXA5 expression and promote cell proliferation, invasion, and angiogenesis [19,29]. To date, there have been few studies of HOXA5 gene expression in human lung cancer [16–19], but their results are contradictory. One study demonstrated that the *HOXA5* gene was less methylated in non-malignant lung tissues than in paired lung cancer tissues [17]. However, another report indicated that HOXA5 expression was higher in squamous cell carcinoma and adenocarcinoma tissues than in non-cancerous tissues [18]. One possible reason for this discrepancy might be that different experimental systems were used in these studies. Our data found that treatment of demethylation agent 5-aza-dC for 5 days increased HOXA5 expression in highly invasive CL1-5 cells but not in CL1-0 cells (Fig 1C). However, the solvent effects of DMSO would partly affect the endogenous HOXA5 expression levels of CL1-0 cells when compared to the QRT-PCR result in Fig 1D. A recent report suggested that HOXA5 could suppress NSCLC cell proliferation partly by regulating p21 expression [30]. However, only a few HOXA5 target genes have been identified [31]; to address this crucial biological problem, the target genes of HOXA5 must be identified systematically.

In this study, DNA microarray analysis was used to investigate the downstream target genes that are differentially regulated in response to HOXA5 expression level. The top ranking pathways influenced by HOXA5 (S2 Table) include cytoskeleton-related pathways such as IP3 signalling, EMT regulation, cell adhesion, and RhoA regulation, which are of particular interest due to their potential roles in lung cancer invasion and metastasis. The data in S2 Table show that the overexpression of HOXA5 decreased the expression of calcium/calmodulin-dependent protein kinase II (CaMKII), a serine/threonine kinase that is regulated by intracellular calcium. The inositol 1,4,5-triphosphate receptor (InsP3R) is an important intracellular calcium release channel in epithelial cells [32]. The type III InsP3R is expressed more strongly in gastric cancer cells than in normal gastric epithelium [33], and its increased expression is also associated with increased aggressiveness of the colorectal carcinoma and decreased long-term survival [32]. Our array and QRT-PCR data also showed that HOXA5 overexpression could decrease the expression level of type III InsP3R. Calcium has been known to serve as an important second messenger and to be involved in the regulation of cell migration [34]. One recent study showed that increased calcium-induced actin assembly is involved in melanoma cell migration [35]. Furthermore, a previous study also demonstrated that the CaMKII β subunit can bind directly to F-actin [36]. All these results suggest that the calcium/InsP3R/CaMKII axis may be involved in lung adenocarcinoma cell migration and that HOXA5 expression can transcriptionally reduce the expression of these genes and inhibit this signalling pathway.

Cell migration is essential for tumor invasion and is a highly integrated multistep process that is initiated by the protrusion of the cell membrane [37]. The protrusive structures of migrating and invading cells, including filopodia we discussed herein, are driven by spatially and temporally regulated actin cytoskeleton polymerisation at the leading edge of the migrating cell [25]. In this study, the enforced expression of HOXA5 downregulated the cytoskeleton remodelling pathway and inhibited filopodia formation, consistent with the previous reports. Moreover, cellular adhesion between tumour cells and elements in their microenvironments is essential for cancer progression. Paxillin is one of the key components within focal adhesions, forming a structural link between the actin cytoskeleton and the extracellular matrix [38]. Recently, paxillin was shown to be essential in actin filament assembly, as well as in cell migration, survival, and morphogenesis [39]. Paxillin expression was found to be higher in more advanced NSCLC (i.e., with higher stages), implying a role for this protein in invasion and metastasis [40]. Our data showed that the overexpression of HOXA5 could downregulate paxillin expression, suggesting that HOXA5 might suppress cell migration and invasion by decreasing paxillin levels and thus affecting actin filament assembly.

A previous study has shown that the actin cytoskeleton plays a pivotal role in the motility of lung cancer cells [40]. The Rho family G proteins are critical regulators of the actin cytoskeleton and are required for cell adhesion, migration, and polarity [41]. GTPase-activating proteins (GAPs) have been suggested as critical negative regulators of Rho GTPase signalling [42]. Our data suggest that the inhibitory effects of GAP on RhoA could partially explain the suppression of migration and invasion in lung cancer cells. In addition, an activator of actin filament nucleation and branching, the actin-related protein (Arp) 2/3 complex, has been reported to act downstream of these three G proteins [43]. The expression of the Arp2/3 complex has been found to be associated with malignant phenotypes in certain types of cancer cells [44]. Moreover, a recent study reported that the Arp2/3 complex could physically associate with RNA polymerase II and participate in transcriptional regulation [45]. These results suggest that the upregulation of Arp2/3 expression might contribute to migration and invasion in malignant cells by controlling actin polymerisation and even targeting gene transcription [46]. The results of our quantitative RT-PCR and functional analyses are consistent with this report. HOXA5 overexpression could decrease the Arp2/3 expression level and suppress migration/invasion.

Furthermore, myosin-X has been known for its localization to the tips of filopodia and its ability to induce filopodia formation [47]. Our immunofluorescence data showed that the expression of myosin-X was significantly decreased in HOXA5-overexpressing cells and led to suppressing the formation of filopodia. However, the expression patterns of fascin, one of the actin binding proteins, showed no dramatic difference between HOXA5-overexpressing and mock control cells. In addition, the cellular morphological change after HOXA5 induction would be partially by regulating E-cadherin and β-catenin expressions (data not shown). It would be very interesting to identify the regulatory mechanisms of HOXA5 in these genes expression in the future studies. Furthermore, p21-activated kinase 1 (PAK1), a serine/threonine protein kinase, has been implicated in the signalling that controls cell polarity, invasion, and actin cytoskeleton organisation [48]. A previous study revealed that A549 cell motility and invasiveness were decreased via c-Crk dephosphorylation after PAK1 silencing [49]. We found that HOXA5 overexpression downregulates PAK1 expression and may suppress the migration and invasive capability of NSCLC cells.

Taken together, although the precise roles of HOXA5 in lung cancer progression remain to be elucidated, we showed here that HOXA5 might act as a suppressor of metastasis during lung tumour progression, at least partly through the inhibition of calcium-mediated actin cytoskeleton polymerisation. However, the detailed molecular mechanisms underlying this inhibition require further investigation. Tetracyclin-inducible system for HOXA5 expression in NSCLC cells would be another good approach to clarify the expression levels and roles of these candidate genes in the future studies. In addition, HOXA5 expression is positively correlated with survival in NSCLC patients, especially those with wild-type EGFR. This result suggests that HOXA5 possibly would serve as a prognostic factor in these NSCLC patients. Nevertheless, the correlation and the regulatory mechanisms between EGFR status and HOXA5 are worthy of further investigation.

## Supporting Information

S1 Fig HOXA5-overexpressing clones suppress NSCLC cell invasion. The invasiveness of CL1-5, HOXA5 (HOXA5 mix, H5, H8, H9, and H14) and mock (Mock mix) transfectants was evaluated by transwell assays. Representative photographs of the invasion assays were shown.
(TIF)

S2 Fig HOXA5-specific siRNA efficiently knockdown HOXA5 mRNA expression. CL1-0 cells were transiently transfected with HOXA5-specific or scramble siRNA and analyzed for HOXA5 mRNA expression by quantitative RT-PCR analysis.
(TIF)

S3 Fig The mRNA expression levels of HOXA5-mediated target genes were upregulated after HOXA5 knockdown. The mRNA expression levels of target genes in HOXA5-specific siRNA transfected cells (HOXA5 siRNA) and scramble siRNA transfectants (Scramble) were measured by quantitative RT-PCR. TATA-binding protein (TBP) was used as an internal control. The data are presented as the mean ± SD of the results from three independent experiments. *, *P*<0.05 compared with the Scramble control.
(TIF)

S1 Table Clinicopathologic characteristics of patients with low and high expression of HOXA5 in the original cohort of 68 non-small cell lung cancer patients. (PDF)

S2 Table HOXA5-associated pathways. (PDF)

S3 Table Gene expression altered by HOXA5. (PDF)
