# Supplementary figures and images for: HOXA5 Inhibits Metastasis via Regulating Cytoskeletal Remodelling and Associates with Prolonged Survival in Non-Small-Cell Lung Carcinoma
Source: PLoS One. 2015 Apr 14;10(4):e0124191. doi: 10.1371/journal.pone.0124191 (PMC4396855; doi:10.1371/journal.pone.0124191)

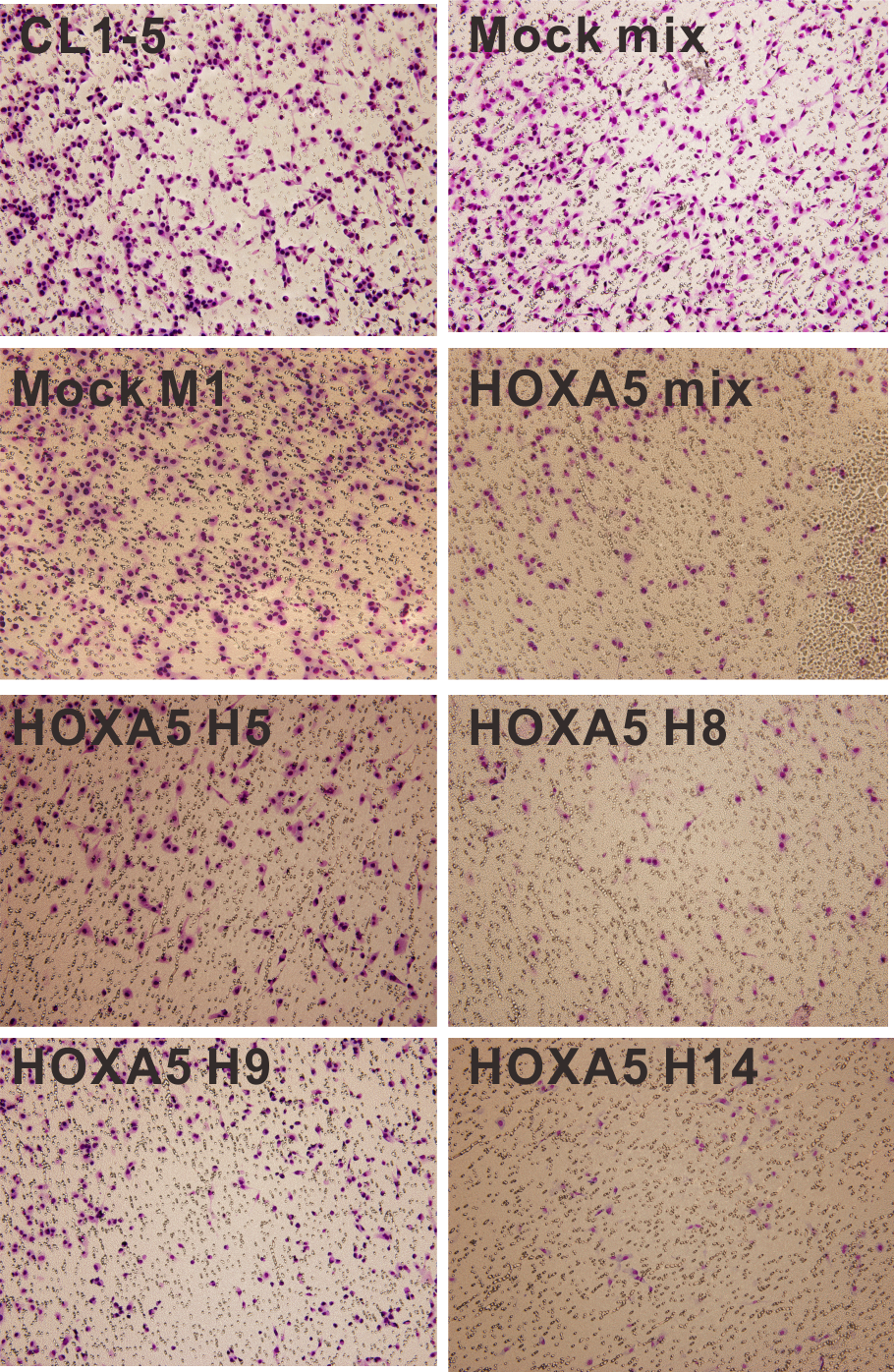

Supplement: S1 Fig — The invasiveness of CL1-5, HOXA5 (HOXA5 mix, H5, H8, H9, and H14) and mock (Mock mix) transfectants was evaluated by transwell assays. Representative photographs of the invasion assays were shown. (TIF) [file pone.0124191.s001.tif]

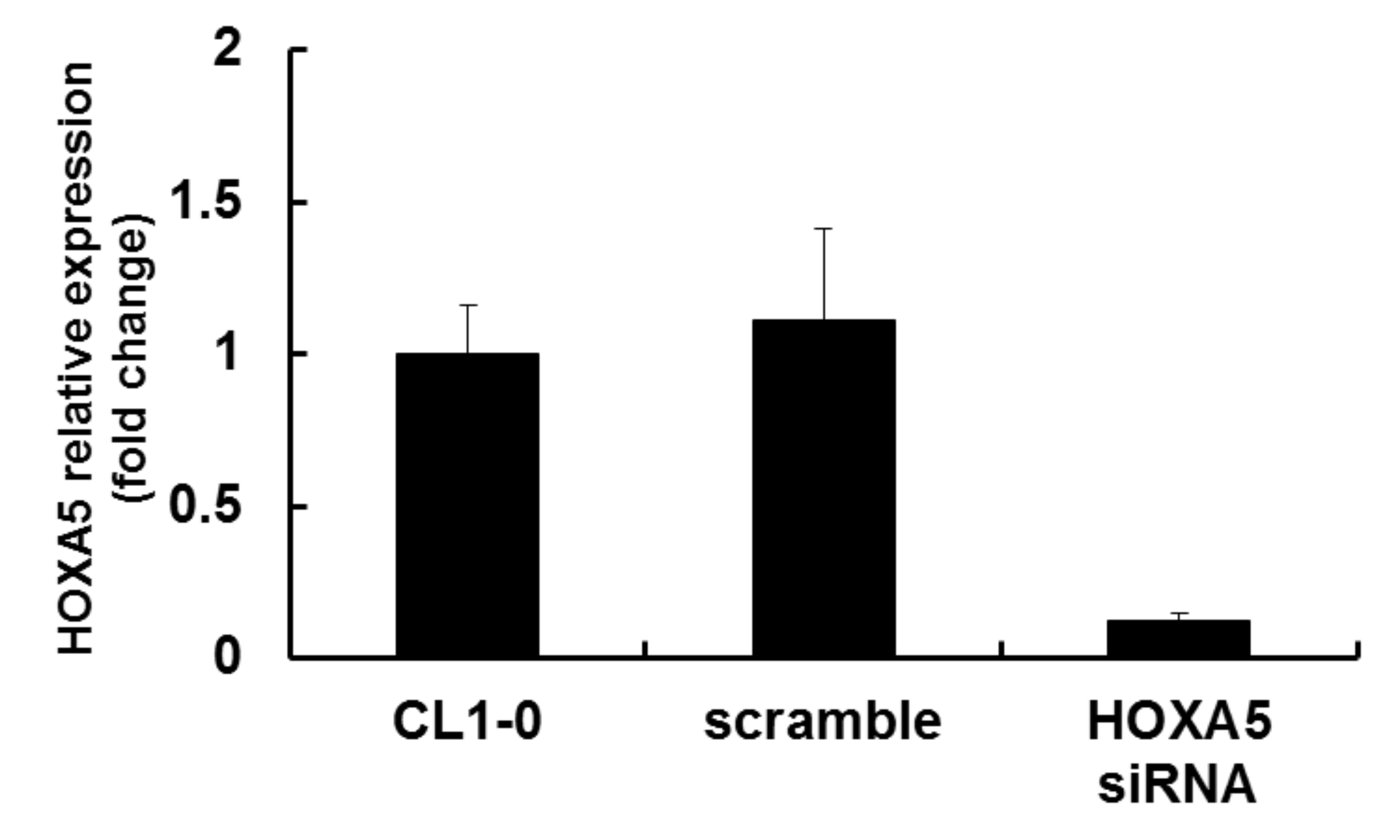

Supplement: S2 Fig — CL1-0 cells were transiently transfected with HOXA5-specific or scramble siRNA and analyzed for HOXA5 mRNA expression by quantitative RT-PCR analysis. (TIF) [file pone.0124191.s002.tif]

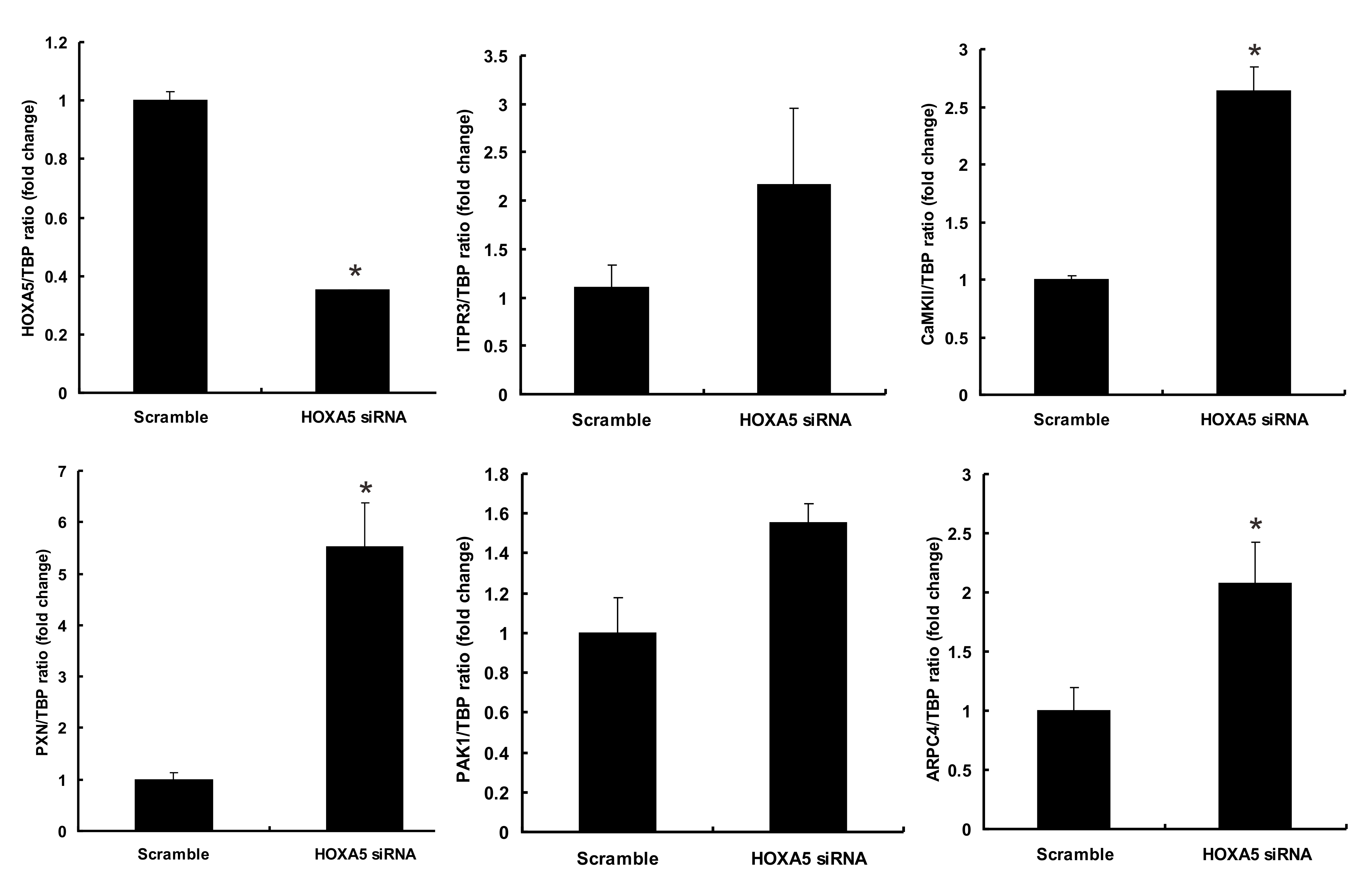

Supplement: S3 Fig — The mRNA expression levels of target genes in HOXA5-specific siRNA transfected cells (HOXA5 siRNA) and scramble siRNA transfectants (Scramble) were measured by quantitative RT-PCR. TATA-binding protein (TBP) was used as an internal control. The data are presented as the mean ± SD of the results from three independent experiments. *, P<0.05 compared with the Scramble control. (TIF) [file pone.0124191.s003.tif]
