# Supplementary material for: HOXA5 Inhibits Metastasis via Regulating Cytoskeletal Remodelling and Associates with Prolonged Survival in Non-Small-Cell Lung Carcinoma
Source: PLoS One. 2015 Apr 14;10(4):e0124191. doi: 10.1371/journal.pone.0124191 (PMC4396855; doi:10.1371/journal.pone.0124191)
Supplement: S1 Table — (PDF) [file pone.0124191.s004.pdf]

S1 Table. Clinicopathologic characteristics of patients with low and high expression of HOXA5 in the original cohort of 68 non-small cell lung cancer patients

**Variable**	**High HOXA5 expression* (20)**	**Low HOXA5 expression* (48)**	***P***	**N (%)**
Age, mean±SD	59.4±14.5	66.9±10.6	0.059	
Gender				
Male	9 (45.0)	33 (68.8)	0.100	42 (61.8)
Female	11 (55.0)	15 (31.3)		26 (38.2)
Cell type				
Adenocarcinoma	11 (55.0)	32 (66.7)	0.415	43 (63.2)
Others	9 (45.0)	16 (33.3)		25 (36.8)
Stage				
I	13 (65.0)	24 (53.3)	0.227	37 (56.9)
II	4 (20.0)	5 (11.1)		9 (13.9)
III	3 (15.0)	16 (35.6)		19 (29.2)

***** 70% percentile for RNA level of *HOXA5* was used as the cutpoint.
