# Supplementary material for: HOXA5 Inhibits Metastasis via Regulating Cytoskeletal Remodelling and Associates with Prolonged Survival in Non-Small-Cell Lung Carcinoma
Source: PLoS One. 2015 Apr 14;10(4):e0124191. doi: 10.1371/journal.pone.0124191 (PMC4396855; doi:10.1371/journal.pone.0124191)
Supplement: S2 Table — (PDF) [file pone.0124191.s005.pdf]

S2 Table. HOXA5-associated pathways

Pathway	*P* value
Development FGFR signalling pathway	1.74 e-9
Cytoskeleton remodeling	2.21 e-9
Development IGF-1 receptor signalling	9.26 e-9
Development EGFR signalling pathway	1.36 e-7
Signal transduction Ip3 signalling	3.60 e-7
Development regulation of EMT	4.35 e-7
Cell adhesion, chemokines and adhesion	2.00 e-7
G protein signalling RhoA regulation pathway	5.26 e-6
