# Supplementary material for: HOXA5 Inhibits Metastasis via Regulating Cytoskeletal Remodelling and Associates with Prolonged Survival in Non-Small-Cell Lung Carcinoma
Source: PLoS One. 2015 Apr 14;10(4):e0124191. doi: 10.1371/journal.pone.0124191 (PMC4396855; doi:10.1371/journal.pone.0124191)
Supplement: S3 Table — (PDF) [file pone.0124191.s006.pdf]

S3 Table. Gene expression altered by HOXA5*

GeneBank accession number	Gene symbol	Gene name	Function	HOXA5/Mock (Fold change)	
				Affymetrix	Real-time RT-PCR
NM_004491	GRLF1/ARHGAP	Rho GTPase activating protein	Signal transduction, transcription corepressor	2.45	1.37
NM_001664	RhoA	ras homolog gene family, member A	Cell adhesion	0.50	0.90
NM_001191	BCL2L1	BCL2-like 1	Anti-apoptosis	0.14	0.77
NM_015981	CaMKII	calcium/calmodulin-dependent protein kinase II	Signal transduction	0.40	0.41
AB209034.1	PXN	Paxillin	Focal adhesion	0.17	0.74
NM_001024959	ARPC4	actin related protein 2/3 complex , subunit 4	Actin filament polymerization	0.44	0.74
NM_001128620	PAK1	p21 protein (Cdc42/Rac)-activated kinase 1	Actin cytoskeleton reorganization	0.44	0.64
NM_002224	ITPR3	inositol 1,4,5-trisphosphate receptor, type 3	Signal transduction	0.23	0.56
NM_001114123	Elk-1	member of ETS oncogene family	Signal transduction	0.42	0.35
NM_001243027	AKT2	v-akt murine thymoma viral oncogene homolog 2	Signal transduction	0.38	0.80

* The differentially expressed genes related to cell growth, adhesion, signal transduction and actin cytoskeleton reorganization, which were identified by Affymetrix oligonucleotide microarray, were validated by SYBR Green real-time RT-PCR.
